# First COVID-19 intra-action review: Experience from Kenya

**DOI:** 10.7189/jogh.13.03043

**Published:** 2023-08-04

**Authors:** Kazuki Shimizu, Nollascus Ganda, Solomon Fisseha Woldetsadik, Juliet Nabyonga-Orem, Miriam Nanyunja, WHO Kenya COVID-19 Incident Management Team

**Affiliations:** 1 World Health Organization Kenya Country Office, Nairobi, Kenya; 2 Emergency Preparedness and Response Programme – Nairobi Hub, World Health Organization Regional Office for Africa, Nairobi, Kenya; 3 Office of the Regional Director, World Health Organization Region Office for Africa, Brazzaville, Congo; 4 Centre for Health Professions Education, Faculty of Health Sciences, North-West University – Potchefstroom Campus, Potchefstroom, South Africa

Since the first report in March 2020, Kenya experienced five waves of coronavirus 2019 (COVID-19), with cases and deaths reported in all 47 counties, amounting to 323 000 and 5600, respectively, in late March 2022 (**Figure 1**). Several public health and social measures were implemented in the country during the pandemic (**Figure 1**, **Table 1**). The vaccination campaign started in March 2021, and the booster campaign in December 2021. By March 2022, 17.6 COVID-19 vaccine doses had been administered [2].

**Figure 1:**
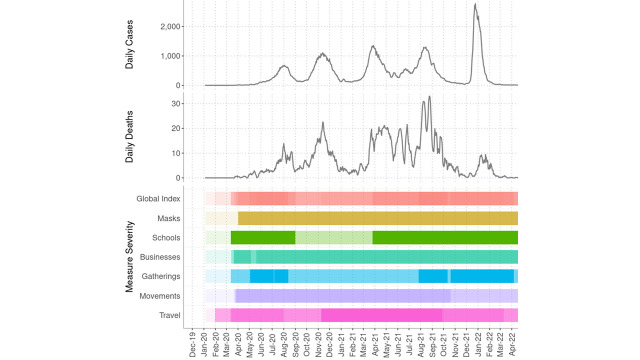
COVID-19 cases, deaths, and measure severity of public health and social measures in Kenya, January 2020 to March 2022. Cases and deaths are based on the date of the report. Measure severity is a composite score calculated from the degree of severity of the response policy and its implementation at a national or sub-national level, based on the global database of public health and social measures applied during the COVID-19 pandemic [1]. It is classified into five levels by density of colours. The Global Index shows the average of the six thematic indicator scores. Masks indicate the intensity of facial coverings and/or mask wearing. School/business closures show the degree of adaptation or closure of schools/businesses. Gatherings demonstrate the degree of limits and restrictions on public and private gatherings. Movement indicates the intensity of restrictions on domestic movement, public transport and stay-at-home orders. Travel demonstrates the degree of international travel restrictions (entry restrictions, quarantining, and testing).

**Table 1:** Key public health and social measures in Kenya, January 2020 to March 2022

Date	Key policy measures
March 2020	Borders closed, no inter-county movement allowed, curfew imposed from 7 PM to 5 AM, religious sites, schools, etc. closed, mandatory testing for all inbound travelers started, mandatory quarantine in government-approved facilities started
June 2020	Curfew shortened from 9 PM to 5 AM
July 2020	Domestic flights resumed, home isolation for asymptomatic and mild cases permitted
August 2020	International flights resumed, religious sites reopened
September 2020	Curfew shortened from 11 PM to 5 AM, bars reopened and restaurants started selling alcohol
November 2020	Curfew extended to 7 PM to 4 AM
March to May 2021	Restriction to/from selected counties imposed, curfew imposed from 8 PM to 4 AM, bars closed and restaurants prohibited to sell alcohol, religious sites, schools, etc. closed
June to July 2021	Curfew extended to 7 PM to 4 AM in 13 counties in Western part of Kenya, gathering and in-person meetings banned
October 2021	Curfew and other measures relaxed
December 2021	Testing for inbound travelers from selected countries started
March 2022	Mandatory mask wearing in open spaces lifted, all in-person worship and indoor meetings at full-capacity resumed when all participants were vaccinated, all quarantines stopped, travelers who completed primary series of vaccines exempted for pre-departure PCR screening test

In responding to epidemics and pandemics, the International Health Regulations (2005) function as an instrument of international law and provide a legal framework that binds member states [3]. Its monitoring and evaluation framework is composed of four components: joint external evaluation, state-party annual reporting, simulation exercise, and after-action reviews (AARs). The latter focus on systematically reviewing functional capabilities and capacities after actual health emergencies. However, as the COVID-19 pandemic necessitated long-term, sector-wide, and sustainable responses and continuous improvement for responding to ongoing and prolonged health emergencies, the World Health Organization (WHO) developed intra-action review (IAR) guidance and tools and encouraged member states to plan and conduct these reviews [4], with the aim of identifying best practices, challenges, and recommendations to improve the ongoing response to COVID-19. Member states in the WHO African Region (AFRO) actively conducted IARs [5,6]. Here we share Kenya’s experience of the first COVID-19 IAR, including the identified challenges, and discuss options to achieve a sustainable COVID-19 response in Kenya.

## INTRA-ACTION REVIEW IN 2021

The first COVID-19 IAR was conducted in Kenya from 31 May to 1 June 2021. Ninety-five from national and county levels participants attended the meeting with the Ministry of Health and multiple stakeholders (such as the Council of Governors, Kenya Prisons Service, WHO, and Centers for Disease Control and Prevention (CDC)). The IAR in Kenya identified best practices and challenges, suggesting recommendations based on nine COVID-19 response pillars:

1. Country-level coordination, planning, and monitoring;
2. Risk communication, community engagement, and infodemic management;
3. Surveillance, case investigation, and contact tracing;
4. Points of entry;
5. National laboratory system;
6. Infection prevention and control;
7. Case management and knowledge sharing about innovations and the latest research;
8. Operational support and logistics in the management of supply chains and workforce resilience;
9. Strengthening essential health services during the COVID-19 outbreak.

Best practices were summarised in four points:

- Strong political will and support at national and county levels, enabling smooth COVID-19 operations and resource allocation;
- Strong coordination and partnership mechanisms;
- Early development and dissemination of guidelines, policies and Standard Operating Procedures (SOPs);
- Utilization of existing structures and resources, such as a legal framework for emergencies, coordination structures, infrastructure, previous capacities developed through training, and existing supply chain management.

Conversely, the key challenges identified were as follows:

- Funding gaps and delayed disbursements, leading to inadequate resources;
- Lack of strong monitoring and evaluation systems;
- Inadequate supply chain management and lack of capacity for local manufacturing [7];
- Inadequate data management systems including paper-based systems;
- Fast-evolving pandemic requiring prompt adaption of systems, infrastructure, guidelines, policies;
- Surge in cases and deaths by waves, with 5180 health worker infections and 40 deaths reported from all counties.

While these findings were largely similar to outcomes in other WHO AFRO countries [5], some strengths in Kenya included the presence of reporting systems at the point of entry, streamlining of sample courier system that leverages existing sample referral systems for polio and influenza programs, relatively strong genomic capacity for respiratory viruses by Kenya Medical Research Institute (KEMRI) Welcome Trust, set-up of biobank and sample repository, as well as local innovation to produce testing ancillary supplies, timely introduction of home-based care, and prioritisation of critical areas for interventions in essential health services.

## CHALLENGES IDENTIFIED BY VARIANTS OF CONCERN, DELTA AND OMICRON

The IAR process was not an evaluation, but an open, honest reflection of key stakeholders on their contribution to the response. It helped with critically reviewing the prolonged COVID-19 response in Kenya and identifying areas for improvement without assigning blame, making honest reflection possible. The findings were successfully shared with multiple partners and senior management decision-makers, helping with the development of a COVID-19 resurgence plan, the review of response policies, guidelines, SOPs, and the application for funding.

However, due to insufficient implementation of IAR recommendations to address the discovered gaps, several challenges identified from mid-2021 still have to be addressed. Since June 2021, the variant of concern (VoC) Delta became dominant in Kenya, and inadequate resources in surveillance, rapid response, and case investigation brought higher SARS-CoV-2 test positivity rate and hindered timely information sharing, adequate risk assessment and projections, effective public health actions, and data-driven decision-making. The high demand for intensive care units (ICUs) and medical oxygen highlighted issues in health service delivery and workforce, including manufacturing, procurement, and supply chain of medical oxygen. Regarding infection prevention and control (IPC), sub-optimal allocation of personal protective equipment (PPEs) contributed to a higher number of COVID-19 patients among health care workers.

The emergence of the Omicron VoC from November 2021 also exposed critical challenges in laboratory pillar to detect new variants. The number of genomic sequencings did not reach the optimal level of over 100 sequences per month [8]. Due to the insufficient availability of reagents and their globally higher demand, there was a delay in implementing the WHO-recommended S-gene target failure approach [9], and Kenya had to rely on the whole-genome sequencing for validation. Furthermore, operations at the point-of-entry control for COVID-19 were overwhelmed due to an increasing number of visitors for the festive season and frequent changes in policies and guidelines. Finally, despite experiences with previous waves, operational support and logistics and supply chain system could not fully cope with a surge of COVID-19 cases whose doubling time was two to three days, unprecedentedly fast. The nature of this surge made it impossible to secure sufficient time to quickly re-conduct stakeholder mapping, mobilise available resources, and achieve better coordination.

## SUSTAINABLE COVID-19 RESPONSE

The IAR was able to identify cross-cutting issues across response pillars. Attendance of health sector partners added value to the overall pandemic preparedness and response. Strong leadership and governance to strengthen incident management structure and response capacity is a key area to achieving an effective coordination at national and sub-national levels. The use of home-based care during COVID-19 has brought positive opportunities and contributed significantly to reduce the burden on health facilities in the country and facilitated the engagement with the community. It is promising that findings from IAR were incorporated into the resurgence plan and were internally reviewed for launching the National Action Plan for Health Security. However, as the COVID-19 pandemic is expected to continue for years, regularly conducting IAR and reviewing the responses across pillars will be beneficial as a monitoring and evaluation tool to continuously improve on identified weak areas for health emergency response. Integrating the vaccine pillar into the next IAR at the national level will enrich the discussion.

These challenges need to be addressed in view of building resilient and sustainable systems for health. Findings from the IAR should inform the review of existing health policies, strategies, and preparedness plans. Despite an initial huge impact, Kenya could gradually mitigate the impact of COVID-19 on essential health services (EHSs), setting a good example [10]. Several rounds of the national pulse survey on continuity of EHSs could help create the monitoring and evaluation framework of EHSs, clarify the capacity of health facilities, and identify indirect effects of COVID-19 on EHSs. These facilitated improving the quality of health services and achieving universal health coverage.

There have also been multiple health emergencies in Eastern Africa, such as outbreaks of yellow fever, cholera, dengue, rift valley fever, and drought in the Horn of Africa. To mitigate their impact on the lives and livelihoods of citizens, Kenya can reinforce and exert its leadership in public health at the regional level. Encouragingly, Kenya will host a second logistic hub which will equip emergency medical personnel, stockpile commodities, and equipment [11] and will support emergency responses in Eastern Africa. Moreover, Kenya was selected as one of six countries to receive the technology for producing mRNA vaccines on the African continent [12] and is expected to acquire the necessary operating procedures and know-how to manufacture mRNA vaccines at scale by following international standards. These initiatives will support the rapid development of new medical countermeasures for known and unknown threats. Concurrently, the investment in an intelligence capacity for health emergencies should not be missed. Encouraging innovation across pillars, including the adaptation of information technology and digitalisation of data management, will bring high-quality data, better analytics, and effective decision-making. Embracing a One Health approach will contribute to protecting citizens from zoonotic, emerging, and re-emerging health threats and building a healthier and more equitable society both at national and regional levels.

In conclusion, we recommend the following:

- The IARs, if conducted with participation of all responders and partners, enable countries to institute corrective measures during the emergency.
- Regular conduction of IAR and review of responses across pillars will help strengthen capacity in health emergency preparedness and response.
- Findings from the IAR must be fully utilised for health initiatives outside of health emergencies, as root causes of challenges are sometimes interlinked.
- The launch of new logistic hub and transfer of technology for vaccine production will elevate Kenya to exert its leadership in public health at the regional level.
